# The scientific production of medical students in Lima, Peru

**DOI:** 10.1016/j.heliyon.2020.e03542

**Published:** 2020-03-19

**Authors:** Diego Urrunaga-Pastor, Christoper A. Alarcon-Ruiz, Paula Heredia, Oscar Huapaya-Huertas, Carlos J. Toro-Huamanchumo, Tania Acevedo-Villar, Lizbeth J. Arestegui-Sánchez, Alvaro Taype-Rondan, Percy Mayta-Tristán

**Affiliations:** a Universidad San Ignacio de Loyola, Unidad de Investigación para la Generación y Síntesis de Evidencias en Salud, Lima, Peru; b Universidad Ricardo Palma, Facultad de Medicina Humana, Lima, Peru; c Universidad Científica del Sur, Carrera de Medicina Humana, Lima, Peru; d Universidad San Luis Gonzaga de Ica, Sociedad Científica de Estudiantes de Medicina de Ica, Facultad de Medicina Humana, Ica, Peru; e Universidad de San Martín de Porres, Facultad de Medicina Humana, Lima, Peru; f Universidad Científica del Sur, Dirección General de Investigación, Desarrollo e Innovación, Lima, Peru

**Keywords:** Social science, Medical Education, Medical

## Abstract

**Background:**

Research is an important undergraduate competence for physicians. However, few studies have assessed the scientific production of medical students in Latin-America. Thus, this study had the objective to assess the rate and characteristics of research publications by undergraduate medical students in 2016, in Lima, Peru.

**Methods:**

This cross-sectional study included all the students of the eight medical schools in Lima (Peru). The medical students included were collected from the registry of the National Medical Examination (taken during their last year of undergraduate studies) in 2016. To evaluate their research publications, systematic searches were performed in Google Scholar and PubMed during August 2018.

**Results:**

We studied data from 1241 medical students (54.2% females) from eight medical schools. 173 (13.9%) students published at least one paper, 102 (8.2%) published at least one original paper, and 30 (2.4%) published at least one original paper in PubMed-Indexed journals. We registered a total of 174 papers authored by medical students, of which 98 (56.3%) were published in Peruvian journals, 128 (73.6%) were published in Spanish, 90 (51.7%) had a medical student as the first author, and 43 (24.7%) had a medical student as the corresponding author. The percentage of students with at least one publication was very heterogeneous across the eight medical schools evaluated (63.6%, 21.4%, 16.8%, 15.1%, 8.2%, 2.0%, 1.9%, and 0.0%).

**Conclusion:**

Among medical students in Lima, one out of seven had published at least one paper, one out of 12 had published at least one original paper, and one out of 40 had published at least one original paper in PubMed-Indexed journals. Scientific production was very heterogeneous across medical schools.

## Teaser

Social science; Medical students; Medical

## 1 Introduction

Physicians need to acquire research competencies in order to provide evidence-based practice and be able to participate in the design and development of research studies [1]. These competencies should optimally be acquired by performing research and publishing research papers during undergraduate training [2, 3, 4, 5]. Accordingly, having performed research during undergraduate training has been associated with better skills in clinical practice and critical reading of scientific papers and clinical practice guidelines [2, 6, 7, 8, 9, 10]. In addition, students that publish research papers may make an important contribution to the scientific production of their universities [11, 12, 13, 14].

In recent years, a growing number of studies are assessing the scientific production of medical students in different countries or regions [7, 15, 16, 17, 18, 19], finding heterogeneous results. For example, 75% of medical students who graduated from Stanford University (USA) in 1991 had published at least one scientific paper [20], 50% of medical students from a research program in Norway had published at least one scientific paper during 2006 [21], and 14% of medical students from the United Kingdom had sent at least one paper to a scientific journal in 2011 [17].

In Latin-America, few studies have assessed the scientific production of a defined group of medical students, and these studies were performed in only one university [22], and in one academic year [23, 24], or among members of scientific societies [25]. Indeed, to our knowledge no study has evaluated and compared the scientific production of students from different medical schools in Latin-America.

Lima is the capital and largest city of Peru, and it has eight medical schools: two receive public funding, and three require a theses dissertation for acquiring a medical degree. Given these heterogeneities across medical schools in Lima, it is relevant to evaluate the scientific production of medical students in each medical school for better understanding of the state of research by medical students. Thus, this study aimed to evaluate the rate and characteristics of research publications by undergraduate medical students in 2016, in Lima, Peru.

## 2 Methods

### 2.1 Design and study population

A cross-sectional study was performed. The study population included graduates from all eight medical schools located in the city of Lima (Peru), who attended their final year in 2016.

In Peru, medical students' study for 7 years, thus the study period of the population included went from 2010 to 2016. Persons that finish high school are able to start medical school regardless their age. It is not needed that medical students publish papers for medical licensing.

The characteristics of each university are described in Table 1. We considered only the medicine schools that had at least one student attending the last undergraduate year during 2016.

**Table 1:** Characteristics of medicine schools included.

Medical School	Year of foundation	Funding	Scientific production in 2016∗	Student volume in class of 2016	Mandatory thesis	Thesis in paper format	Research courses credits∗∗
UNMSM	1856	Public	291	146	No	No	4/308
UPCH	1961	Private	408	126	Yes	No	3/302
UNFV	1966	Public	19	105	No	No	6/308
USMP	1984	Private	97	334	No	No	7/308
UPSJB	1997	Private	8	135	No	No	5/315
UCSUR	1998	Private	49	148	No	No	5/335
URP	1998	Private	34	170	Yes	No	5/332
UPC	2007	Private	153	77	Yes	Yes	10/341

UCSUR: Universidad Científica Del Sur, USMP: Universidad de San Martin de Porres, UNFV: Universidad Nacional Federico Villarreal, UNMSM: Universidad Nacional Mayor de San Marcos, UPCH: Universidad Peruana Cayetano Heredia, UPC: Universidad Peruana de Ciencias Aplicadas, UPSJB: Universidad Privada San Juan Bautista, URP: Universidad Ricardo Palma.

∗ Search in Scopus, in December 17th, 2018.

∗∗ Information extracted from: Taype-Rondan A, Huaccho-Rojas J, Pereyra-Elias J, Mejia CR, Mayta-Tristan P. [Research courses' characteristics in Peruvian medical schools]. Archivos de Medicina. 2015; 11(2):1–7.

We obtained the list of all the names of the medical students from medical schools of Lima (Peru) taking the National Examination Board (ENAM, in Spanish) in 2016, from the website of the Peruvian Association of Faculties of Medicine (ASPEFAM, in Spanish) (http://www.aspefam.org.pe/). This list is published on free access.

The ENAM is an exam applied annually by the ASPEFAM to medical students during their last year of study [26]. As noted, undergraduate medical training in the included universities lasts seven years. This exam is required to apply for residency and to apply for the Rural and Urban Marginal Health Service (which in turn allows physicians to work for the public sector in Peru) [26, 27].

### 2.2 Search strategy

During August 2018, systematic searches were conducted in Google Scholar and PubMed to determine the scientific production of the medical students included in the study. Google Scholar is considered the search engine that includes the largest amount of biomedical literature available on the Internet [28].

To build the terms of the search strategy, we used all the possible combinations of the names and surnames (“paternal” and “paternal-maternal”) of each student, adding the terms [Peru] or [Lima] separately. This strategy was used in previous studies separately, similar to previous studies [29, 30]. Subsequently, we reviewed each paper manually to avoid the inclusion of possible cases of homonymy. We select a paper into the present study following the inclusion criteria: 1) The name (or any variant in their order) of the student was registered as a name author of the paper, 2) The student author's affiliation was one of the eight medical schools from Lima; 3) The paper was published from 2010 to 2016 (period of time that students attended the medical school). We included research articles that had a journal number publication date prior to the final of the students' university studies. We select this cut-off point because not all articles evaluated presented a reception or publication date in their manuscript.

Two researchers independently carried out the systematic search and manual review of the publications of each medical student. Later, a third researcher resolved the discordant cases. We considered any paper published in a scientific journal to be a scientific publication (e.g., original articles with IMRaD format, reviews, case reports and letters to the editor). Books, technical reports, theses, posters, or scientific congresses abstracts were excluded.

### 2.3 Variables

We collected the variables taking into account two different units of analysis: medical students and papers.

#### 2.3.1 Medical student variables

The following variables were collected for each medical student: gender (male or female), medical school in which the undergraduate studies were carried out, having published at least one paper in a scientific journal (yes or no), having published at least one original paper in a scientific journal (yes or no), having published at least one original paper in a scientific journal indexed in PubMed (yes or no), and the year of first publication. The paper was considered to be an "original" paper if it had the following sections: introduction, methods, results, and discussion; or the equivalents. All the papers published up to 2016 were registered (according to the year corresponding to the issue of the journal).

#### 2.3.2 Paper variables

The following variables were collected for each paper: name and country of the journal in which it was published (Peru, Latin-America, others), language of the paper (Spanish, English, Spanish and English) year to which the issue of the journal corresponded, type of article (original, letter to the editor, clinical case reports, or others), if the paper was indexed in PubMed (yes or no), if the paper had a medical student as first author (yes or no), if the paper had a medical student as the corresponding author (yes or no), article design (descriptive, analytic, experimental, quasi-experimental), and the article topic (basic sciences, clinical, public health/epidemiology, medical education).

### 2.4 Statistical analysis

Descriptive analysis was carried out using absolute and relative frequencies for the categorical variables. Data analysis was performed using Stata v14.0.

### 2.5 Ethical considerations

The present study was evaluated and approved by the Ethics Committee of the Universidad Científica del Sur (UCSUR) in Lima, Peru. In addition, following data collection, the names of the medical students were replaced by codes protecting the identity of the participants prior to analysis.

## 3 Results

We studied 1241 medical students (54.2% were female) from 8 medical schools, of which 173 (13.9%) had published at least a paper, 102 (8.2%) had published at least one original paper, and 30 (2.4%) had published at least one original paper in PubMed (Table 2). The range of papers published by student varied from 0 to 36.

**Table 2:** Characteristics of evaluated students (N = 1241).

Characteristics	N (%)
Gender	
Male	569 (45.9)
Female	672 (54.2)

University	

UCSUR	148 (11.9)
USMP	334 (26.9)
UNFV	105 (8.5)
UNMSM	146 (11.8)
UPCH	126 (10.2)
UPC	77 (6.2)
UPSJB	135 (10.9)
URP	170 (13.7)

Number of papers	

None	1068 (86.1)
1	125 (10.1)
2	25 (2.0)
3	14 (1.1)
4 or more	9 (0.7)

Have published at least one original paper	

No	1139 (91.8)
Yes	102 (8.2)

Have published at least one original paper in Pubmed	

No	1211 (97.6)
Yes	30 (2.4)

Year of first publication	

2011	10 (5.8)
2012	19 (11.0)
2013	18 (10.4)
2014	32 (18.5)
2015	56 (32.4)
2016	38 (22.0)

UCSUR: Universidad Científica Del Sur, USMP: Universidad de San Martin de Porres, UNFV: Universidad Nacional Federico Villarreal, UNMSM: Universidad Nacional Mayor de San Marcos, UPCH: Universidad Peruana Cayetano Heredia, UPC: Universidad Peruana de Ciencias Aplicadas, UPSJB: Universidad Privada San Juan Bautista, URP: Universidad Ricardo Palma.

**Figure 1:**
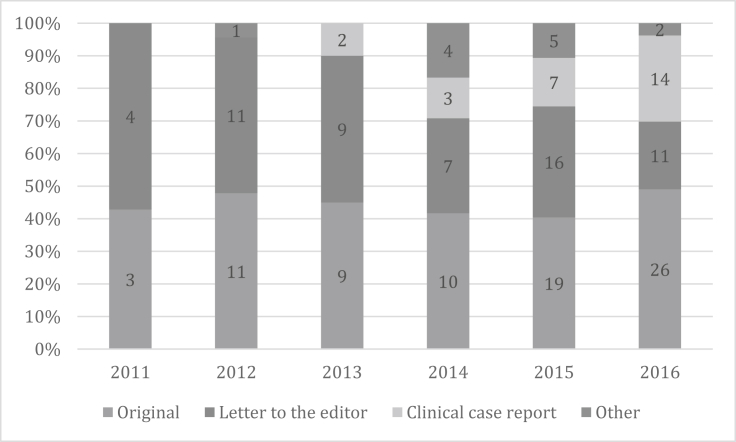
Trends in the type of article published by year.

According to the type of article, letters to the editor tended to diminish over time, clinical reports tended to increase, while original and other types of articles did not present any defined trend (Figure 1).

**Figure 2:**
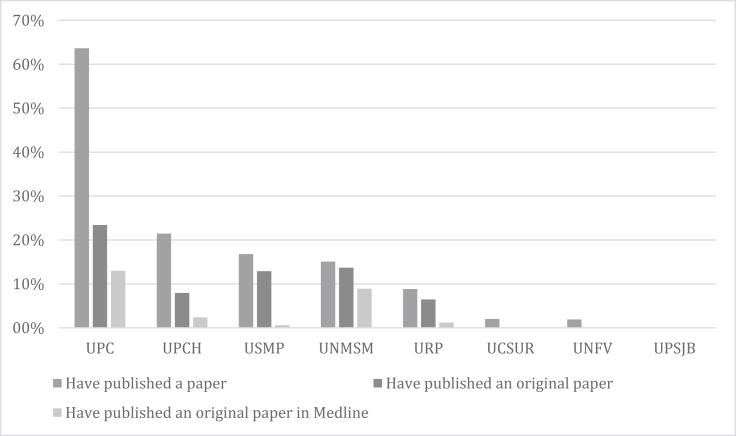
Scientific production of medical students by medical school. UPC: Universidad Peruana de Ciencias Aplicadas (N = 77), UPCH: Universidad Peruana Cayetano Heredia (N = 126), USMP: Universidad de San Martin de Porres (N = 334), UNMSM: Universidad Nacional Mayor de San Marcos (N = 146), URP: Universidad Ricardo Palma (N = 170), UCSUR: Universidad Científica Del Sur (N = 148), UNFV: Universidad Nacional Federico Villarreal (N = 105), UPSJB: Universidad Privada San Juan Bautista (N = 135).

The percentage of students that had published at least one paper was higher in the Universidad Peruana de Ciencias Aplicadas (UPC) (63.6%), followed by the Universidad Peruana Cayetano Heredia (UPCH) (21.4%), the Universidad de San Martín de Porres (USMP) (16.8%), the Universidad Nacional Mayor de San Marcos (UNMSM) (15.1%), the Universidad Ricardo Palma (URP) (8.2%), the Universidad Científica del Sur (UCSUR) (2.0%), the Universidad Nacional Federico Villarreal (UNFV) (1.9%) and the Universidad Peruana San Juan Bautista (UPSJB) (0.0%). However, the percentage of students that had published at least one paper in PubMed was higher for the UPC (13.0%), followed by UNMSM (8.9%) and UPCH (2.4%) (Figure 2).

We registered a total of 174 papers authored by medical students, of which 98 (56.3%) were published in Peruvian journals, 128 (73.6%) were published in Spanish, 53 (30.5%) were published in 2016, 78 (44.8%) were original articles, 58 (33.3%) were letters to the editor, 81 (46.6%) were indexed to PubMed, 90 (51.7%) had a medical student as the first author, 43 (24.7%) had a medical student as the corresponding author, 41 (52.6%) original articles had an analytic design, and 36 (46.1%) presented a public health/epidemiology topic. Any of the articles followed a qualitative methodology (Table 3).

**Table 3:** Characteristics of papers published by students (N = 174).

Characteristics	N (%)
Journals	

Peruvian journals	98 (56.3)
Revista Peruana de Medicina Experimental y Salud Pública	21 (12.1)
Horizonte Medico	16 (9.2)
Revista Médica Herediana	11 (6.3)
Revista de la Facultad de Medicina de la Universidad Ricardo Palma	9 (5.2)
Anales de la Facultad de Medicina	7 (4.0)
CIMEL	7 (4.0)
Revista de Neuro-psiquiatría	5 (2.9)
Other Peruvian journals	21 (12.1)
Latin-American	24 (13.8)
Others	53 (30.5)

Language	

Spanish	128 (73.6)
English	45 (25.9)
Spanish and English	1 (0.6)

Year according to journal	

2011	7 (4.0)
2012	23 (13.2)
2013	20 (11.5)
2014	24 (13.8)
2015	47 (27.0)
2016	53 (30.5)

Type of article	

Original	78 (44.8)
Letter to the editor	58 (33.3)
Case report	26 (14.9)
Other	12 (6.9)

Indexed in Pubmed	

No	93 (53.5)
Yes	81 (46.6)

Has as first author a medical student	

No	84 (48.3)
Yes	90 (51.7)

Has as corresponding author a medical student	

No	131 (75.3)
Yes	43 (24.7)

Article design∗	

Descriptive	22 (28.2)
Analytic	41 (52.6)
Experimental	10 (12.8)
Quasi-experimental	5 (6.4)

Article topic∗	

Basic sciences	14 (18.0)
Clinical	13 (16.7)
Public health/Epidemiology	36 (46.1)
Medical education	15 (19.2)

∗ Includes only original articles.

## 4 Discussion

Our study found that, among medical students in Lima, one out of seven had published a paper, one out of 12 had published an original paper, and one out of 40 had published an original paper in PubMed. The students evaluated published 174 articles, of which just over half were published in Peruvian journals, three out of four were published in Spanish, slightly less than half were original papers, more than half were published between 2015 and 2016, and almost half had a medical student as the first author. Regarding medical schools, great heterogeneity was found with respect to the percentage of students who had published papers, with the UPC presenting the highest percentage.

### 4.1 Scientific production of medical students

In our study, 13.9% of students had published at least one paper, although this percentage was heterogeneous across medical schools (from 0% to 63.6%). Likewise, previous studies in Peruvian medical schools have reported a heterogeneous prevalence of publication of the undergraduate thesis: 2.7% in a private medical school in Lima during the period 2000–2009 [31], 17.6% in another private medical school between 2000-2003 [32], and 11.8% in a public medical school in Lima from 1998-2008 [33].

On the other hand, according to previous studies, the prevalence of medical faculty members having published a paper in a scientific journal in Peru was 56.2% for scientific research professors in 2011 [30], 24% for medical school deans in 2014 [34], and 14.3% for research Vice Chancellors in 2016 [35]. While it is expected for these professionals to have a much greater scientific production than the medical students, the differences are not so big, especially taking into account that 63.6% of medical students from a certain medical school had published a paper during their medical studies. This could be indicative of a change in mindset, from a poor publication culture to a new paradigm.

The percentage of students that had published at least one paper in our study (13.9%) was higher than that of another study carried out in 2009 in 190 Colombian medical students from the University of Cali. In this study the prevalence of scientific production of medical students during their career was 3.2% [22]. Our results are similar to a study conducted in 2009 in medical students belonging to a scientific society of medical students (SOCEM, for its initials in Spanish) in 30 medical schools in Colombia which found that 17% had published at least one paper in their lives [25] (students who do not belong to the SOCEM are expected to have a lower scientific production [24]).

In addition, our findings are similar to reports from outside Latin America: a prospective 2-year follow-up cohort conducted at a medical school in Sweden between 2010-2012 found that 15.5% of medical students had published at least one article within two years after completing their research project [7], and to another study conducted in six medical schools in the Netherlands which found that 14.5% of medical students had published at least one scientific article indexed in the Web Of Science (WOS) during their last three academic years [16].

### 4.2 Paper characteristics

Most of the papers found in the present study were published in Peruvian journals, similar to what has previously been described on evaluating the papers from a scientific congress of medical students in Peru, in which all were published in Peruvian journals [29]. Of the ten most popular journals for publishing, four belong to the medical schools included in the study, possibly because for students it is easier to publish in the scientific journals of their own medical school. However, since these journals are not indexed in international databases such as PubMed or Scopus, students should be encouraged to publish in foreign journals to increase the visibility of their studies.

Only one out of four articles were published in English, which may be due to the lack of advanced knowledge of English writing among Peruvian medical students [36]. This could prevent students from publishing in high impact journals with greater international visibility, restricting most studies to a local audience.

Publications of letters to the editor diminished over time. This may be because the students during their first years focus on papers that do not need much methodological knowledge, as letters to the editor. Moreover, the proportion of clinical reports increased over time, which is expected since these papers need clinical knowledge and access to patients. On the other side, original articles and other types of articles did not present a defined trend.

Regarding article design, less than a third of studies were descriptive, which suggest that students are either performing their own inference analyses or are collaborating with other researchers that perform such analyses. With respect to the article topic, just under half of the studies were in the public health/epidemiology, and the less common category was clinical studies. This may be due to the greater ease of conducting public health/epidemiology studies, while permissions for clinical studies tend to be harder to get. Almost half of the articles included had a medical student as the first author, agreeing with a review article published in 2013, which evaluated the scientific production of medical students worldwide in Scopus and PubMed and reported that 48.6% of the 356 papers found had a medical student as the first author [15]. However, at a local level, it was found that in 17.9% of the articles published and indexed in SciELO-Peru during 1997–2005 a medical student was the first author [37], although it was difficult to compare studies since this study was performed in only one database. The high percentage of first authorship could be due to great cooperative work between mentors and students. In these cases, mentors include the student in their research projects, and the student carries out most of the research work, thereby receiving the first authorship and the mentor assumes the senior and correspondent position [38].

### 4.3 Medical school characteristics

We found that the UPC, UPCH, UNMSM, and the USMP led the percentage of medical students with at least one paper. However, scientific productions seem to depend on different factors per medical school.

The UPC has developed and applied a *structural academic intervention*, which includes a sequence of research courses aimed to develop competencies in research [39]. Professors who publish during their research studies [30] have two to five research projects/theses each. Students are required to send a paper to an indexed scientific journal as part of their studies [40], and the possibility to acquire the medical doctor degree is achieved through the publication of a scientific article in an indexed journal [41].

On the other hand, UPCH students have a scientific promotion environment. The UPCH is the medical school with the highest scientific production in Peru [42, 43]. Many research institutes and excellence centers with prolific and experienced researchers actively cooperate with undergraduate students from this university [44, 45, 46]. In addition, the UPCH has the highest economic funding in the country for their undergraduate students [12].

Lastly, the UNMSM [47], USMP [48], and the URP [49], each have active scientific student associations which aim to train and advise medical students in basic methodological research and statistical skills and promote scientific production. These activities are usually organized in cooperation with a scientific advisor experienced in research, who is not necessarily part of the medical school staff. The remaining medical schools (UCSUR, UNFV, and UPSJB) do not have any of these interventions, yet.

### 4.4 Implications

We found that one out of seven medical students had published a paper, which is of note considering that Peru has a poor scientific production.

However, this higher publication rate mainly occurs in Lima, since medical schools located in the rest of the country may have a lower scientific production [14, 37], being an important issue, which should be evaluated. In addition, we observed a great heterogeneity among universities, possibly due to the different strategies used in each medical faculty. Medical schools interested in improving their scientific production should assess the *structural academic intervention* performed by the UPC and determine which approaches are the most adequate for their particular contexts.

### 4.5 Limitations and strengths

This study has some limitations: 1) Only one cohort of each medical school was evaluated, which does not represent all the students of each medical school; 2) The sample studied was not representative at a national level but rather was made to evidence the current situation of scientific production of medical students in Peru.

Nevertheless, this is the first study to analyze the scientific production of students in a representative population of several medical schools in Lima, Peru, thereby providing an overview of student research in our country.

### 4.6 Conclusions

Of a total of 1241 students, the prevalence of scientific production was 14.0%. Moreover, one out of 12 students had published an original article, and one out of 40 had published an original article in PubMed. Of the 174 articles found, just over half were published in Peruvian journals, three out of four were published in Spanish and slightly less than half were original articles. We recommend implementing interventions in Peruvian medical schools in order to increase the scientific production of undergraduate medical students.

## Declarations

### Author contribution statement

D. Urrunaga-Pastor, C. A. Alarcon-Ruiz: Conceived and designed the experiments; Performed the experiments; Analyzed and interpreted the data; Contributed reagents, materials, analysis tools or data; Wrote the paper.

P. Mayta-Tristán, A. Taype-Rondan: Conceived and designed the experiments; Analyzed and interpreted the data; Wrote the paper.

P. Heredia, O. Huapaya-Huertas, C. J. Toro-Huamanchumo, T. Acevedo-Villar, L. J. Arestegui-Sánchez: Performed the experiments; Contributed reagents, materials, analysis tools or data; Wrote the paper.

### Funding statement

This research did not receive any specific grant from funding agencies in the public, commercial, or not-for-profit sectors.

### Competing interest statement

The authors declare the following conflict of interests: Diego Urrunaga-Pastor has studied at the Universidad de San Martín de Porres, and is a master degree student at Universidad Peruana Cayetano Heredia. Lizbeth J. Arestegui-Sánchez has studied at the Universidad de San Martín de Porres. Christoper A. Alarcon-Ruiz has studied at Universidad Ricardo Palma, and is a master degree student at Universidad Peruana Cayetano Heredia. Paula Heredia has studied at Universidad Ricardo Palma, and is a master degree student at Universidad de San Martín de Porres. Oscar Huapaya-Huertas has studied at the Universidad Científica del Sur and the Universidad Peruana Cayetano Heredia; and has worked for the Universidad Científica del Sur. Carlos J. Toro-Huamanchumo has studied at the Universidad de San Martín de Porres and Universidad Peruana Cayetano Heredia; and has worked for the Universidad de San Martín de Porres, the Universidad Peruana de Ciencias Aplicadas and the Universidad Peruana Cayetano Heredia. Alvaro Taype-Rondan has studied at the Universidad de San Martín de Porres and Universidad Peruana Cayetano Heredia; and has worked for the Universidad de San Martín de Porres, the Universidad Peruana Cayetano Heredia, and the Universidad Científica del Sur. Percy Mayta-Tristán has studied at the Universidad Nacional Mayor de San Marcos; and has worked for the Universidad Peruana de Ciencias Aplicadas, the Universidad Peruana Cayetano Heredia and the Universidad Científica del Sur.

### Additional information

No additional information is available for this paper.
